# Assessing the effects of Ang-(1-7) therapy following transient middle cerebral artery occlusion

**DOI:** 10.1038/s41598-019-39102-8

**Published:** 2019-02-28

**Authors:** M. M. C. Arroja, E. Reid, L. A. Roy, A. V. Vallatos, W. M. Holmes, S. A. Nicklin, L. M. Work, C. McCabe

**Affiliations:** 1 Glasgow Experimental MRI Centre (GEMRIC), Institute of Neuroscience & Psychology, College of Medical, Veterinary & Life Sciences, University of Glasgow, Glasgow, UK; 2 BHF Glasgow Cardiovascular Research Centre, Institute of Cardiovascular & Medical Sciences, College of Medical, Veterinary & Life Sciences, University of Glasgow, Glasgow, UK

## Abstract

The counter-regulatory axis, Angiotensin Converting Enzyme 2, Angiotensin-(1-7), Mas receptor (ACE2/Ang-1-7/MasR), of the renin angiotensin system (RAS) is a potential therapeutic target in stroke, with Ang-(1-7) reported to have neuroprotective effects in pre-clinical stroke models. Here, an extensive investigation of the functional and mechanistic effects of Ang-(1-7) was performed in a rodent model of stroke. Using longitudinal magnetic resonance imaging (MRI) it was observed that central administration of Ang-(1-7) following transient middle cerebral artery occlusion (MCAO) increased the amount of tissue salvage compared to reperfusion alone. This protective effect was not due to early changes in blood brain barrier (BBB) permeability, microglia activation or inflammatory gene expression. However, increases in NADPH oxidase 1 (Nox1) mRNA expression were observed in the treatment group compared to control. In order to determine whether Ang-(1-7) has direct cerebrovascular effects, laser speckle contrast imaging (LSCI) was performed to measure dynamic changes in cortical perfusion following reperfusion. Delivery of Ang-(1-7) did not have any effect on cortical perfusion following reperfusion however; it showed an indication to prevent the ‘steal phenomenon’ within the contralateral hemisphere. The comprehensive series of studies have demonstrated a moderate protective effect of Ang-(1-7) when given alongside reperfusion to increase tissue salvage.

## Introduction

In the UK, more than 152,000 people will suffer a stroke accounting for approximately 40,000 deaths every year^1^. Intravenous (IV) alteplase is the main line of therapy for acute ischaemic stroke, however, its eligibility is limited due to the narrow therapeutic time window (<4.5 hr) and safety concerns^2^. Recently, endovascular thrombectomy has shown to be an effective strategy with an extended therapeutic window (6 to 24 hr post stroke), particularly in large proximal occlusions^3–5^. This new line of therapy has reinvigorated the stroke community with the possibility of translation of adjunctive therapies alongside recanalisation that can act to increase efficacy of these approaches. In the present study, we have investigated the potential of Ang-(1-7) as an adjunctive treatment following recanalisation.

The classical axis of the RAS has been widely implicated in ischaemic stroke pathology through becoming over-activation of the Angiotensin Converting Enzyme/Angiotensin II/Angiotensin II receptor type I (ACE/Ang II/AT_1_R) arm. The role of the classical RAS axis in ischaemic stroke pathology has been shown in knockout (KO) studies where AT_1_R KO mice exhibited a larger penumbra volume and improved cerebral blood flow (CBF) within the ischaemic core and penumbra^6^. As a result, AT_1_R antagonists (candesartan, olmesartan, valsartan and irbesartan) have been tested and shown to reduce infarct volume, improve perfusion, inhibit BBB breakdown and reduce oxidative stress, inflammation and microglia activation following experimental stroke^7–11^. Moreover, a recent study demonstrated that an Ang II vaccine is neuroprotective following ischaemic stroke, thus, suggesting that targeting the RAS is a promising therapeutic approach^12^. While the role of the ACE/AngII/AT_1_R axis is relatively well established in experimental models of stroke, increasing evidence now suggest that the RAS offers an endogenous cerebroprotective mechanism through the activation of the counter-regulatory RAS axis composed of ACE2/Ang-(1-7)/MasR.

Ang-(1-7) is an endogenous constituent of the brain and its receptor Mas is expressed in neurons, endothelial cells, astrocytes and microglia^13–16^. Central administration of Ang-(1-7) has been shown to reduce infarct size in rat models of middle cerebral artery occlusion (MCAO), an effect that has been suggested to be mediated at least in part by inhibiting central inflammation and maintaining integrity of the BBB as well as being MasR dependent^15,17–22^. Following transient MCAO, Ang-(1-7) delivery was shown to prevent BBB breakdown by leading to tight junction preservation through metalloproteinase 9 (MMP9) downregulation and enhancement of its inhibitor, tissue inhibitor of metalloprotease 1 (TIMP1)^23^. In endothelin-1 (ET-1) induced MCAO, Ang-(1-7) therapy attenuated infarct size and neurological deficit due to a proposed reduction in inducible nitric oxide synthase (iNOS) at acute stages of injury^17^. Similarly, in permanent MCAO models, Ang-(1-7) treatment was suggested to decrease infarct volume as a result of NF-κB suppression and inhibition of interleukin 1 beta (IL-1β), interleukin 6 (IL-6) and cyclooxygenase 2 (COX2) expression 24 hr post MCAO^19^. Ang-(1-7) is hypothesized to exert its effects by directly acting on MasR present on microglia at acute stages of injury following MCAO and preventing the upregulation of pro-inflammatory mediators IL-6, IL-1β, iNOS and cluster of differentiation 11 b (CD11b) whilst stimulating the generation of anti-inflammatory cytokine, interleukin 10 (IL-10)^15,18^. Additionally, reports suggest that Ang-(1-7) may exert pro-angiogenic effects or act through a vasodilatory effect and therefore, lead to an increase in CBF following cerebral injury^20,24–26^. Still, the latter proposed effect is controversial and remains elusive^17,20^.

Although mounting evidence implicates the ACE2/Ang-(1-7)/MasR axis as a potential therapeutic target following ischaemic stroke, the majority of pre-clinical studies have been performed using a permanent model of MCAO or the ET-1 induced MCAO model, which results in gradual reperfusion. This is in contrast to the abrupt reperfusion that would be observed following endovascular thrombectomy an effect that is mimicked with the intraluminal filament model of transient MCAO^27^. Consequently, the aim of this study was to elucidate the neuroprotective potential of Ang-(1-7) as an adjunctive post-stroke therapy at acute and subacute stages of injury following transient MCAO. Three broad aims were addressed in order to determine the functional and mechanistic effects of Ang-(1-7). Firstly, to investigate the therapeutic potential of post-reperfusion administration of Ang-(1-7) on the extent of tissue salvage using MRI. Second, to investigate the effects of Ang-(1-7) on BBB breakdown in the acute phase post-stroke using contrast enhanced MRI imaging. Third, to investigate the impact of systemic delivery of Ang-(1-7) on the cerebrovascular response post-reperfusion using laser speckle contrast imaging (LSCI). Finally, in an attempt to dissect potential mechanisms, gene expression levels for inflammatory/oxidative stress markers were examined and the effect of Ang-(1-7) on microglia number/activation studied.

## Results

### Study 1: Mortality & Exclusions

A total of 10 animals died prior to the 7 day end point (6 vehicle and 4 Ang-(1-7)). From all surviving animals, 10 were excluded from analysis either due to the intracerebroventricular (ICV) cannula not being within the cerebral ventricle for drug delivery or an incomplete occlusion of the MCA was observed on MR angiography. Furthermore, systolic BP was not obtained for one vehicle animal at 7 days post MCAO due to animal stress during the procedure.

### Ang-(1-7) treatment increases tissue salvage following reperfusion at 7 days post MCAO

**Figure 1:**
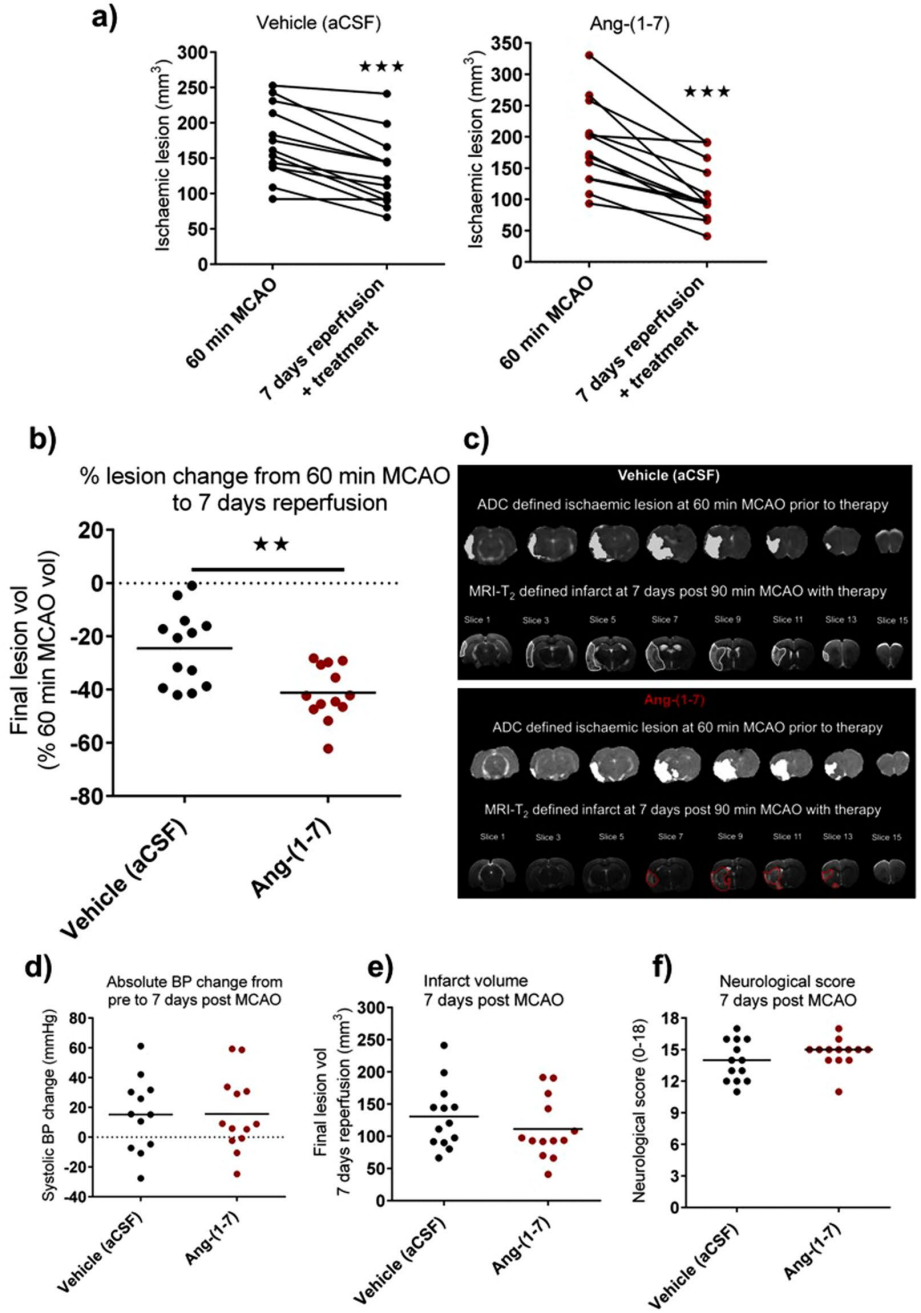
Ang-(1-7) treatment for 7 days significantly attenuates lesion growth from 60 min MCAO lesion. (**a**) Ischaemic lesion evolution from 60 min MCAO (prior to therapy) to 7 days post MCAO along with therapy for each individual animal in both vehicle (1 μl/hr aCSF; n = 13) and Ang-(1-7) (1.1 nmol/hr; n = 13) treated groups. Ischaemic lesion and reperfusion extent varies amongst rats. (**b**,**c**) Ang-(1-7) ICV therapy along with reperfusion significantly decreases ischaemic lesion evolution from 60 min MCAO to 7 days post MCAO compared to vehicle. **(d)** Therapy effects are independent of systolic BP induced alterations from pre to 7 days post MCAO (n = 12–13 per group). **(e**,**f)** Final infarct volume or neurological score evaluated at day 7 only did not differ amongst groups (n = 13 per group). Data are presented as mean ± S.D; **P < 0.01; ***P < 0.001; unpaired Student’s t test.

Baseline lesion volume for the treatment groups at 60 min post MCAO (prior to treatment starting) was: vehicle (artificial cerebrospinal fluid (aCSF)); 171.8 ± 50.8 mm^3^ and Ang-(1-7); 187.1 ± 67.7 mm^3^ (Fig. 1a). At day 7 post-reperfusion and following treatment infusion, infarct volume significantly decreased for all animals in vehicle (aCSF) (130.6 ± 50.7 mm^3^; P < 0.001) and Ang-(1-7) (111.3 ± 47.4 mm^3^; P < 0.001) groups (Fig. 1a). The extent of lesion variability can be observed in both treatment groups during MCAO as well as the extent of decrease following reperfusion. Therefore, to account for the variability observed and examine therapeutic effects alongside reperfusion, each animal was used as its own control. This allowed calculation of the extent of tissue salvage following reperfusion with/without Ang-(1-7).

Ang-(1-7) along with reperfusion increased the extent of tissue salvage at day 7 when compared to vehicle (41.2 ± 10.2% vs 24.5 ± 14.1% reduction in baseline lesion volume, P < 0.01) (Fig. 1b,c). This outcome was independent of any effects of Ang-(1-7) on changes in systolic blood pressure (BP) over the 7 day time course (change in systolic BP from baseline to day 7: Vehicle, 15.2 ± 25 mmHg vs Ang-(1-7), 15.58 ± 25 mmHg; P > 0.05) (Fig. 1d). Interestingly, when assessing final infarct volume at day 7 only, there were no significant differences between Vehicle and Ang-(1-7) groups (130.6 ± 50.7 mm^3^ vs 111.3 ± 47.4 mm^3^; P > 0.05) (Fig. 1e). Accordingly, there were no differences in neurological deficit at day 7 when assessed by the 18-point neurological score (Fig. 1f).

### Study 2: Ang-(1-7) does not influence BBB breakdown 24 hr post MCAO

Two animals in the vehicle group died 24 hr post MCAO and three rats were excluded due to the ICV cannula not being situated in the ventricle for drug infusion. Five animals were excluded from analysis of BBB breakdown due to unsuccessful tail vein injection of contrast agent.

**Figure 2:**
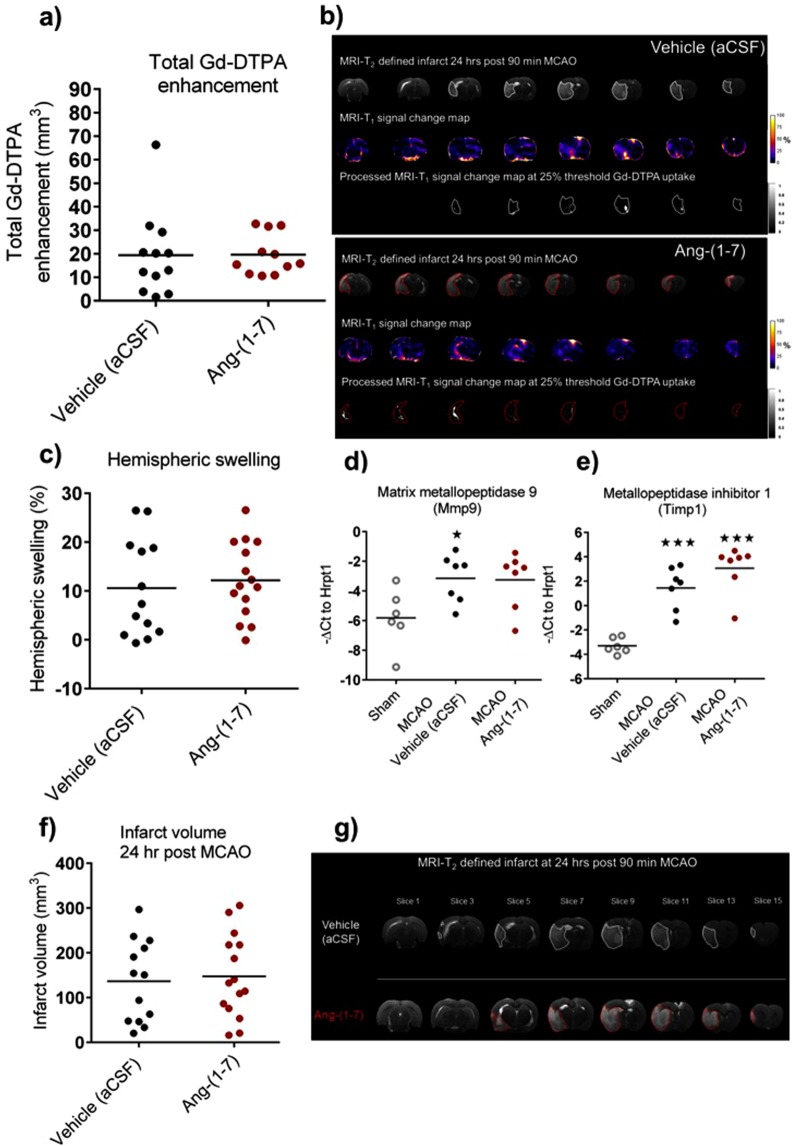
Ang-(1-7) does not influence BBB breakdown 24 hr post MCAO. (**a**,**b**) Gd-DTPA enhancement volume across the ipsilateral hemisphere did not differ between Ang-(1-7) (1.1 nmol/hr; n = 11) and control rats (aCSF; n = 12). **(c)** Ang-(1-7) therapy (n = 15) did not alter hemispheric swelling volume compared to Vehicle (aCSF; n = 13). **(d)** Gene expression levels for BBB breakdown marker, Mmp9, were significantly upregulated in MCAO vehicle group (n = 7) and nearly reaching significance in Ang-(1-7) treated rats (n = 7) compared to sham rats (n = 6). Ang-(1-7) did not alter Mmp9 mRNA levels compared to vehicle treated rats. **(e)** Gene expression values for Mmp9 inhibitor, Timp1, were significantly upregulated in MCAO groups (n = 7 per group) compared to sham animals (n = 6) without Ang-(1-7) induced effects compared to control. **(f**,**g)** Ang-(1-7) ICV therapy (n = 15) for 24 hr did not alter infarct volume in comparison to vehicle treated rats (n = 13). Data are presented as mean ± S.D. *P < 0.05; ***P < 0.001; unpaired Student’s t test. Gene expression data were compared using one-way ANOVA with Tukey’s posthoc test; *P < 0.05; ***P < 0.001 compared to sham.

The 18-point neurological score at 24 hr post MCAO confirmed neurological deficit in both groups with no significant differences observed (Supplementary Fig. 2a). At 24 hr post MCAO; RARE-T_2_ MRI demonstrated significant brain swelling in both groups (Supplementary Fig. 2b). The uptake of gadolinium-diethylenetriamine penta-acetic acid (Gd-DTPA) contrast agent was calculated for both groups in order to determine BBB leakage. Compared to vehicle (aCSF) treated animals, Ang-(1-7) therapy did not alter Gd-DTPA enhancement volume (19.7 ± 8.7 mm^3^ vs 19.4 ± 7.7 mm^3^, P > 0.05) (Fig. 2a,b). Similarly, Gd-DTPA enhancement expressed as percentage of infarct did not differ amongst groups (Supplementary Fig. 2c). Additionally, Ang-(1-7) did not decrease hemispheric swelling compared to control animals (12.2 ± 7.7% vs 10.6 ± 10.1%, P > 0.05) (Fig. 2c). These data demonstrated that at 24 hr post MCAO; the BBB may only be partially open since Gd-DTPA uptake volume was small in many animals and much smaller than the T_2_ weighted infarct.

Ang-(1-7) is suggested to decrease Mmp9, a marker for BBB breakdown, and increase the gene expression of its inhibitor, Timp1^23^. Consequently, Mmp9 and Timp1 gene expression levels were evaluated for both MCAO treated groups and sham animals in peri-infarct regions (Fig. 2d,e). Compared to sham rats, Mmp9 mRNA levels were significantly enhanced following MCAO in vehicle (aCSF) (−3.1 ± 1.6 vs −5.8 ± 2.0, vehicle vs sham, P = 0.04) and although not quite reaching statistical significance, levels were also increased in Ang-(1-7) treated rats (−3.3 ± 1.9 vs −5.8 ± 2.0, Ang-(1-7) vs sham, P = 0.053) (Fig. 2d), confirming BBB breakdown. Similarly, Timp1 was significantly upregulated compared to sham in both vehicle (2.1 ± 2.5 vs −3.3 ± 0.6, vehicle vs sham, P < 0.001) and Ang-(1-7) (3.1 ± 1.9 vs −3.3 ± 0.6, Ang-(1-7) vs sham, P < 0.001) treated animals (Fig. 2e). Ang-(1-7) treatment did not change infarct volume in comparison to vehicle treated animals (147.6 ± 92.7 mm^3^ vs 136.4 ± 91.4 mm^3^, P > 0.05) (Fig. 2f,g).

### AT_2_R and MasR mRNA expression is altered at 7 days following MCAO in the peri-infarct regions

**Figure 3:**
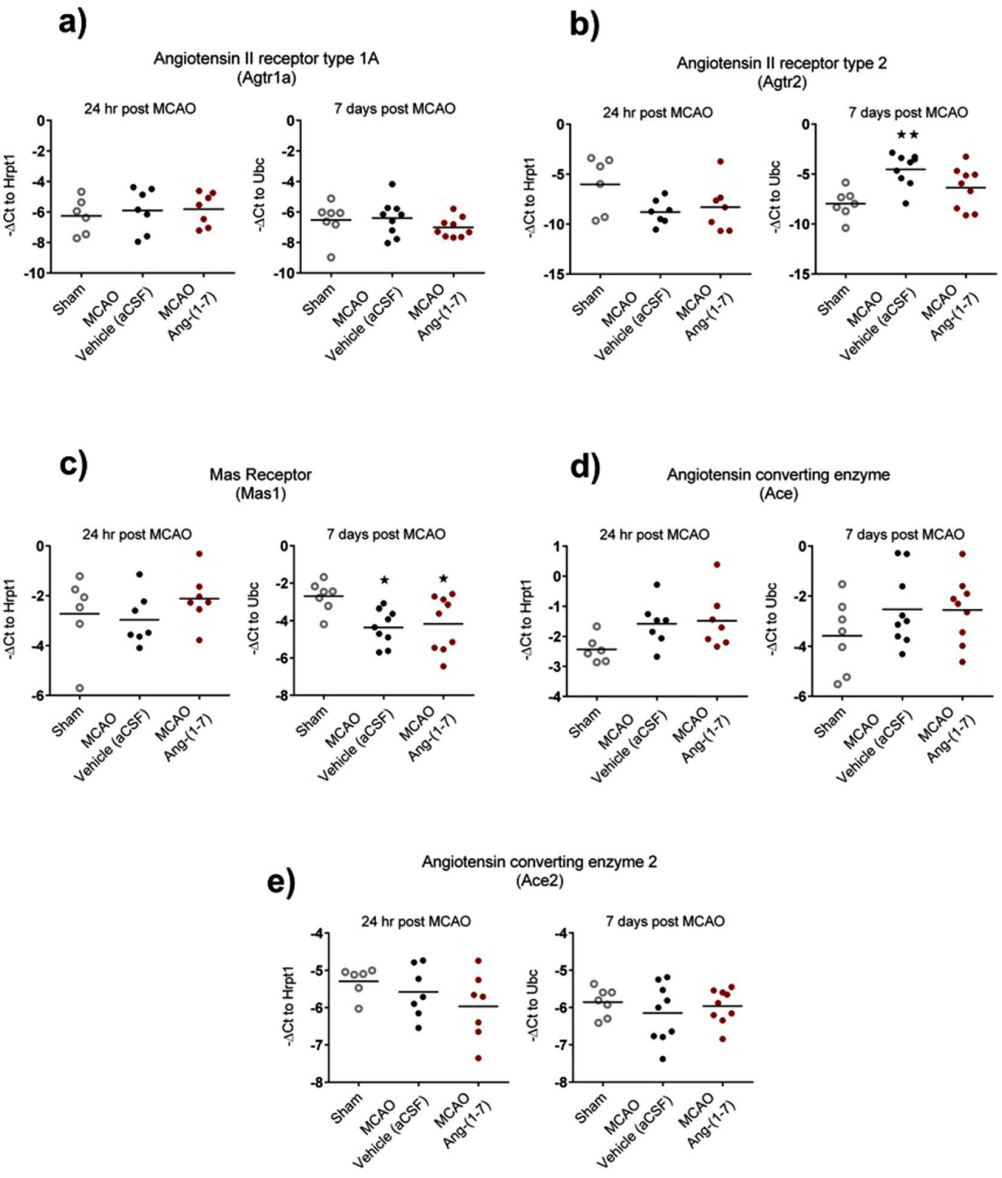
Ang-(1-7) does not influence RAS mRNA expression at 24 hr or 7 days post MCAO in peri-infarct regions. (**a**) Atgr1a levels were comparable to sham at 24 hr and 7 days post MCAO without Ang-(1-7) treated effects. **(b)** Atgr2 mRNA expression did not alter at 24 hr post MCAO when compared to sham. However, at 7 days reperfusion, gene expression were significantly upregulated in MCAO-vehicle group. **(c)** Mas1 was comparable to sham levels 24 hr post MCAO; yet, at 7 days post MCAO, levels were significantly downregulated in MCAO groups without Ang-(1-7) mediated effects. **(d**,**e)** Ace and Ace2 were comparable to sham at 24 hr and 7 days post MCAO. Data displays values for sham (n = 6-7); MCAO-Vehicle (aCSF) (n = 7–9) and MCAO-Ang-(1-7) (n = 7–9) treated rats. Data are presented as mean ± S.D. **P < 0.01 compared to sham; one-way ANOVA with Tukey’s posthoc test.

AT_1_R (Fig. 3a), AT_2_R (Fig. 3b), MasR (Fig. 3c), ACE (Fig. 3d) and ACE2 (Fig. 3e) mRNA levels were measured at 24 hr and 7 days post MCAO in peri-infarct brain regions obtained from sham, MCAO-vehicle and MCAO-Ang-(1-7) ICV treated rats. AT_1_R (Atgr1a) levels were comparable to sham at 24 hr and 7 days following MCAO in both treatment groups (Fig. 3a) and similarly, AT_2_R (Agtr2) levels at 24 hr were unchanged (Fig. 3b). In contrast, AT_2_R (Atgr2) expression was significantly upregulated at day 7 following MCAO in vehicle treated rats compared to sham (−4.5 ± 1.6 vs −8.0 ± 1.4, P < 0.01) whereas AT_2_R (Atgr2) levels in Ang-(1-7) treated rats were not statistically different between sham and vehicle treated groups (Fig. 3b). At 24 hr post MCAO MasR expression was unchanged in both treatment groups when compared to sham treated rats. However, at day 7 post MCAO, MasR (Mas1) levels significantly decreased in both vehicle (aCSF) (−4.4 ± 1.0 vs −2.7 ± 0.8, P = 0.02) and Ang-(1-7) (−4.2 ± 1.5 vs 2.7 ± 0.8, P = 0.04) treated groups compared to sham treated animals (Fig. 3c). No changes in either Ace or Ace2 mRNA levels were detected at 24 hr and 7 days post MCAO when compared to sham treated rats (Fig. 3d,e).

### Ang-(1-7) significantly attenuates Nox1 mRNA expression at 7 days post MCAO

**Figure 4:**
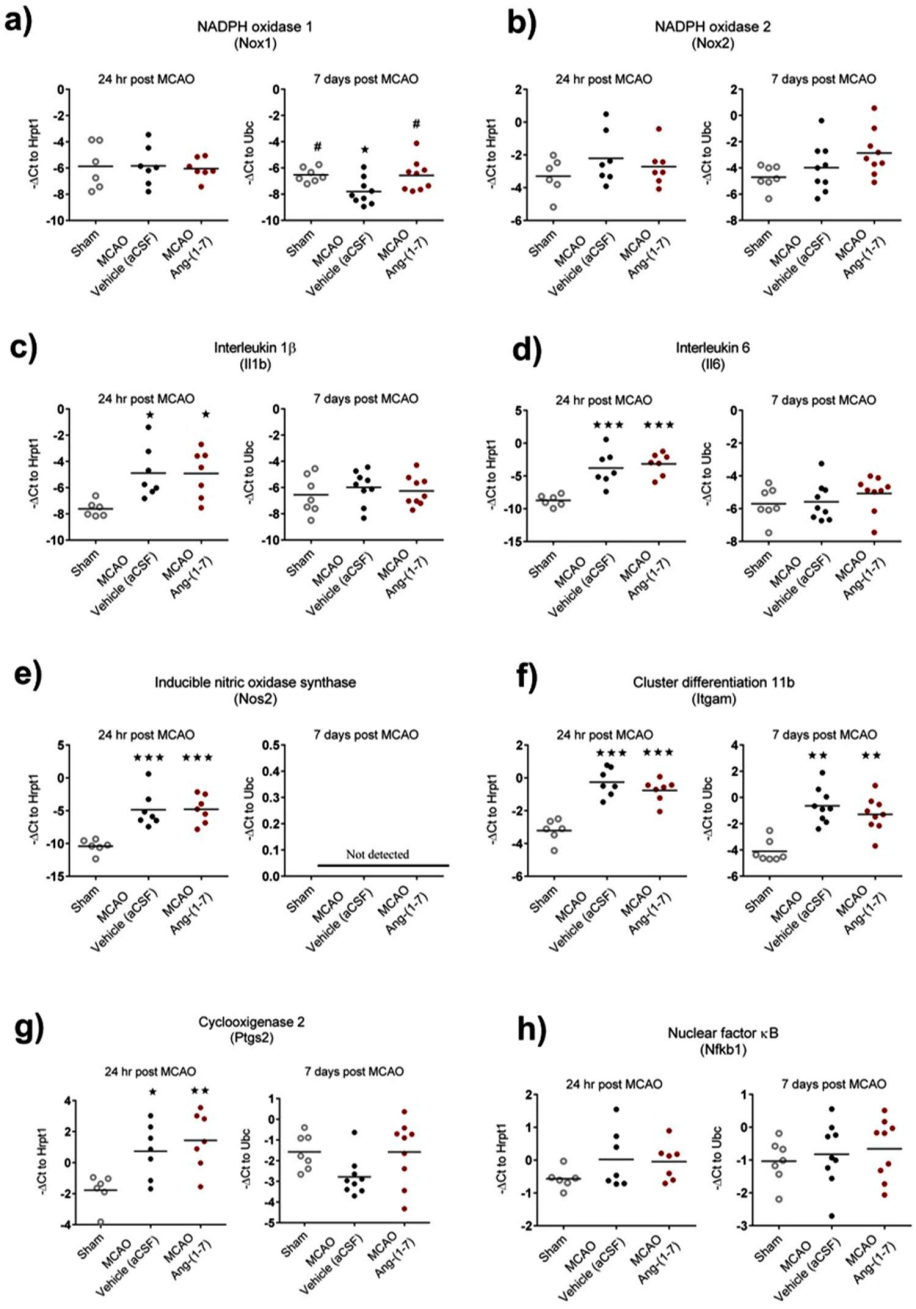
Ang-(1-7) significantly attenuates Nox1 gene expression to sham levels 7 days post MCAO in peri-infarct regions. (**a**) Nox1 was comparable to sham at 24 hr for MCAO-Vehicle and MCAO-Ang-(1-7) treated groups. Conversely, at 7 days post MCAO, Nox1 was significantly decreased in MCAO-Vehicle group with Ang-(1-7) treatment attenuating the expression to sham levels (P < 0.05). **(b)** Nox2 expression did not alter at 24 hr or 7 days post MCAO when compared to sham. **(c**–**h)** Il1b, Il6, Nos2, Itgam, Ptgs2 or Nfkb1 expression was not influenced by Ang-(1-7) therapy at 24 hr and 7 days post MCAO. Data displays values for sham (n = 6–7); MCAO-Vehicle (aCSF) (n = 7–9) and MCAO-Ang-(1-7) (n = 7–9) treated rats. Data are presented as mean ± S.D. *P < 0.05 compared to sham; ^#^P < 0.05 compared to MCAO-Vehicle; one-way ANOVA with Tukey’s posthoc test.

Given the proposed anti-oxidative and anti-inflammatory effects of Ang-(1-7), we investigated mRNA expression levels in peri-infarct brain tissue of genes involved in regulating oxidative stress and inflammation (Fig. 4a–h). The oxidative stress markers Nox1 and Nox2 were unchanged when compared to sham treated rats at 24 hr post MCAO (Fig. 4a,b). However, at 7 days post MCAO, Nox1 mRNA expression was significantly reduced in vehicle treated rats compared to sham (−7.8 ± 1.0 vs −6.5 ± 0.6; P = 0.04) and this reduction was attenuated in Ang-(1-7) treated rats (−6.6 ± 1.2 vs −7.8 ± 1.0, Ang-(1-7) vs vehicle; P = 0.03) (Fig. 4a). In contrast, Nox2 levels were unchanged at day 7 days post MCAO (Fig. 4b). In terms of markers of inflammation there were no differences between Ang-(1-7) and vehicle treated rats for Il1b, Il6, Nos2, Itgam (also known as CD11b), Ptgs2 (also known as COX2) or nuclear factor κB (NF-κB; (Nfkb1)) at 24 hr or 7 days post MCAO (Fig. 4c–h). Furthermore, Ang-(1-7) did not influence mRNA expression for M2 microglia/macrophage type markers C-C motif chemokine 22 (CCL22), arginase 1 (Arg1), cluster of differentiation 163 (CD163) or transforming growth factor β1 (TGF-β1) compared to vehicle treated animals (Supplementary Fig. 3a–d).

### Ang-(1-7) does not alter microglia activation at 24 hr or 7 days post MCAO

**Figure 5:**
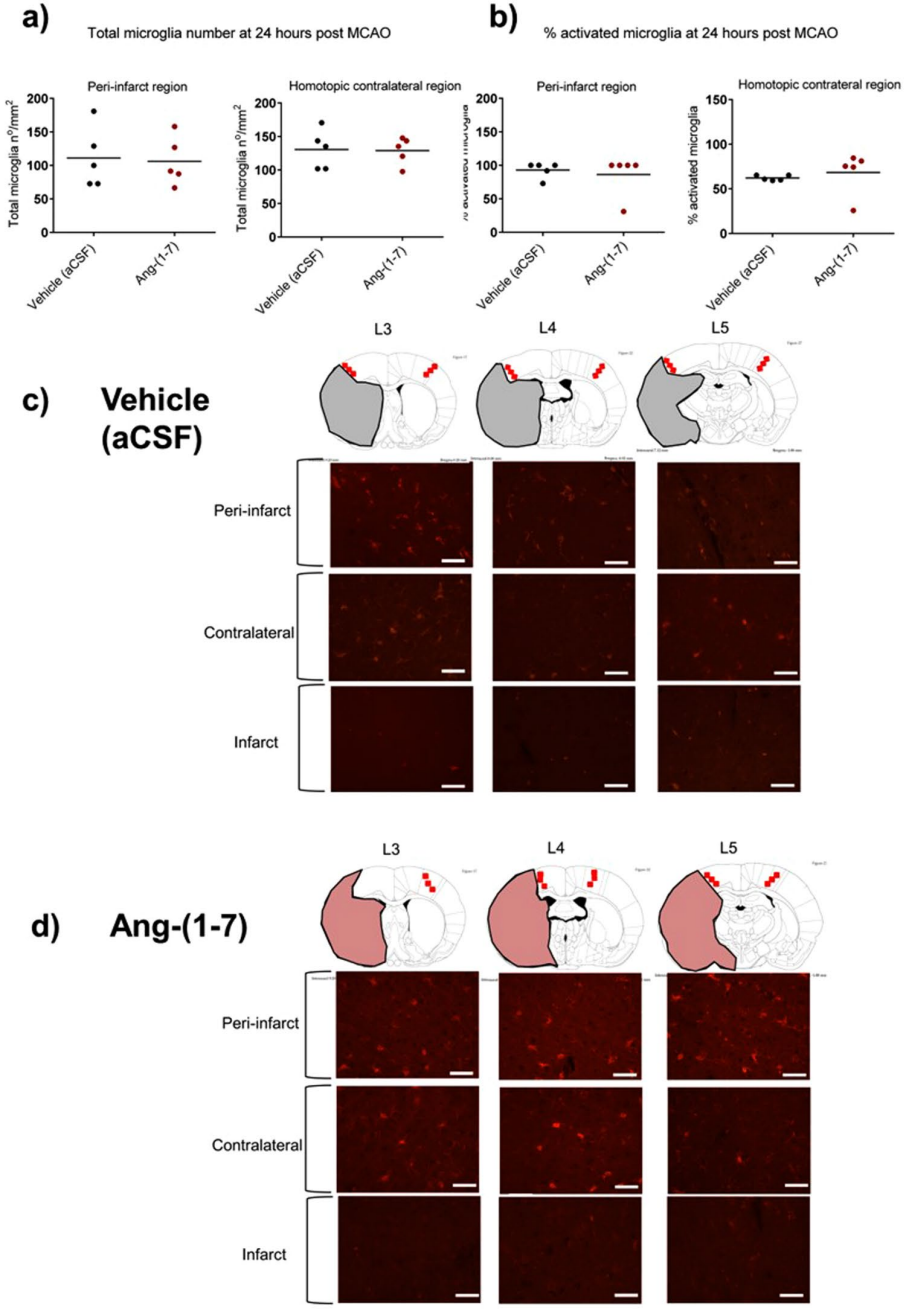
Ang-(1-7) therapy has no effect on microglia number or phenotype at 24 hr following MCAO. (**a**,**b**) Ang-(1-7) ICV therapy (1.1 nmol/hr; n = 5) did not change IBA1^+^ microglia total number or % activated cells within peri-infarct or homotopic contralateral regions 24 hr post MCAO compared to Vehicle (aCSF; n = 6) rats. **(c**,**d**) Representative images of IBA1^+^ microglia staining for vehicle and Ang-(1-7) treated median animals within the peri-infarct, homotopic contralateral and infarct regions. Line diagrams adapted from The Rat Brain in Stereotaxic Coordinates by G. Paxinos and C. Watson, 1998.

IBA1^+^ microglia was assessed in the peri-infarct and homotopic contralateral regions at 24 hr and 7 days post MCAO. Ang-(1-7) did not significantly alter total microglia number in the peri-infarct (106.1 ± 36.3 n°/mm^2^ vs 111.1 ± 45.4 n°/mm^2^, P > 0.05) or homotopic contralateral regions (129.0 ± 20.3 n°/mm^2^ vs 130.6 ± 29.3 n°/mm^2^, P > 0.05) when compared to Vehicle (aCSF) treated rats (Fig. 5a). Similarly, Ang-(1-7) therapy did not influence the % activated microglia in the peri-infarct region (86.2 ± 30.8% vs 93.0 ± 11.7%, P > 0.05) or homotopic contralateral regions (62.2 ± 2.9% vs 68.3 ± 24.1%, P > 0.05) when compared to control animals (Fig. 5b). Figures 5c,d illustrate the IBA1 immunofluorescence from the median rat from each treatment group at 24 hr post MCAO. At day 7 post MCAO microglia staining was qualitatively assessed and demonstrated no differences between treatment groups (Supplementary Fig. 4a–d).

### Study 3: Systemic infusion of Ang-(1-7) attenuates the ‘steal’ phenomenon in the non-ischaemic hemisphere following reperfusion

Next, the effects of Ang-(1-7) systemic administration on the cerebrovasculature following reperfusion were investigated. Seven rats were excluded: 5 rats failed to reperfuse after removal of the filament, 1 rat had a partial occlusion of the middle cerebral artery (MCA) and 1 rat had excessive bleeding over the skull surface affecting imaging (Supplementary Fig. 5). Infusion of Ang-(1-7) had no effect on mean arterial blood pressure (MABP) compared to vehicle rats (Supplementary Fig. 6a) whilst PaO_2_, PaCO_2_ and pH values were maintained stable amongst animals (Supplementary Table 1).

**Figure 6:**
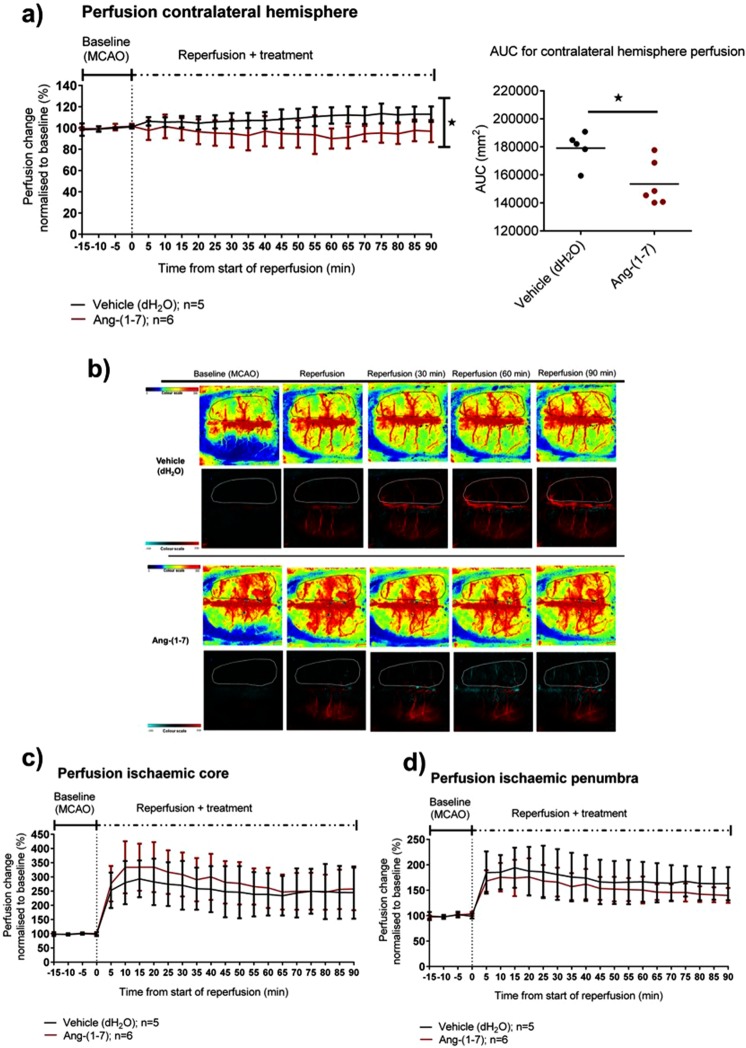
Ang-(1-7) IV therapy significantly attenuates cerebral perfusion increase in the contralateral hemisphere over time. (**a**,**b**) Ang-(1-7) IV infusion therapy (5 nmol/hr; n = 6) significantly attenuated cerebral perfusion increase observed in the contralateral hemisphere in control treated animals (dH_2_O; n = 5) (P < 0.05). (**c**,**d**) Ang-(1-7) therapy did not change perfusion within the ischaemic core and ischaemic penumbra regions during reperfusion. Data were analysed with mean AUC comparisons followed by unpaired Student’s t test; *P < 0.05 was deemed as significant. Data are expressed as mean ± S.D. % change from baseline.

In the contralateral hemisphere, cerebral perfusion over the first 90 min following reperfusion increased by 11.8 ± 8.1% in vehicle treated rats whereas Ang-(1-7) treatment attenuated this increase in perfusion (4.6 ± 10.8%). Comparison of the area under the curve (AUC) values (first 90 min post reperfusion) demonstrated a statistically significant difference between vehicle and Ang-(1-7) treated rats (P = 0.01) (Fig. 6a,b). In the ischaemic core, reperfusion led to a marked increase in perfusion in vehicle (dH_2_O) and Ang-(1-7) treated groups peaking at approximately 15 min post reperfusion, 194.6 ± 63.9% vs 233.1 ± 84.6% respectively (Fig. 6c). Ang-(1-7) treatment showed a trend to enhance perfusion compared to the control group, however, comparison of AUC values indicated that differences were not significant when compared to the vehicle group (P > 0.05). In the ischaemic penumbra region of interest (ROI), reperfusion resulted in an increase in perfusion that peaked at 15 min, 95.2 ± 42.1% vs 74.1 ± 30.2% for vehicle and Ang-(1-7) groups, respectively. During the course of reperfusion, Ang-(1-7) did not impact ischaemic penumbra perfusion when compared to Vehicle as determined by comparison of mean group AUC values (P > 0.05) (Fig. 6d). Furthermore, Ang-(1-7) did not change the frequency of peri-infarct depolarisations (PIDs) following reperfusion compared to control animals (0.5 ± 0.55 vs 0.2 ± 0.5 (P > 0.05)), (Supplementary Fig. 6b). Overall, Ang-(1-7) did not alter cortical perfusion in ipsilateral hemisphere or the incidence of PIDs, however, it attenuated the ‘steal phenomena’ towards the contralateral hemisphere.

## Discussion

In this comprehensive series of studies investigating the role of Ang-(1-7) administration following stroke it was demonstrated that: 1. Central administration increases tissue salvage following reperfusion at 7 days post MCAO; 2. Ang-(1-7) does not alter early permeability of the BBB or microglia activation at 24 hr post MCAO; 3. Ang-(1-7) administration does not alter brain RAS, inflammatory or oxidative stress gene expression; however it attenuates the decrease in Nox1 gene expression and 4. Systemic infusion of Ang-(1-7) attenuates the ‘steal’ phenomenon observed following reperfusion in the non-ischaemic hemisphere.

The counter-regulatory RAS axis, ACE2/Ang-(1-7)/MasR, has received recent focus and has been suggested to be a potential therapeutic target following ischaemic stroke^15,17,19^. Here, the therapeutic potential of Ang-(1-7) administered following stroke was examined at acute and subacute stages using imaging techniques. The experiments conducted had strict exclusion criteria through the use of MRI that confirmed the following: correct ICV cannula placement for delivery of Ang-(1-7), complete occlusion of the MCA following filament insertion and successful recanalisation of the MCA following filament removal.

The use of DWI-MRI immediately following MCAO (60 min) allowed baseline lesion volume during MCAO to be calculated prior to reperfusion and/or initiation of treatment. This is important as it allowed us to investigate the influence of reperfusion with or without Ang-(1-7) on the change in lesion volume over time within individual animals thereby increasing the statistical power of our studies. Interestingly, when assessing final infarct volume at day 7, there was no significant effect of Ang-(1-7) treatment when compared to vehicle treatment. This likely reflects the inherent variability observed in infarct volume with the transient intraluminal filament model of MCAO and highlights the limitations of assessing a single time point in drug studies. One of the major advantages of the present study was the fact that each animal had baseline lesion volume assessed prior to reperfusion ± treatment. It was observed that there is considerable variability in the size of lesion during MCAO even with confirmation of complete occlusion of the MCA with MR angiography. This may be due inter-animal variability in the extent of collateral vessel supply^28^. For all animals, reperfusion at 90 min resulted in a decrease in the baseline lesion volume by day 7 with Ang-(1-7) treatment resulting in a greater extent of tissue salvage by day 7 when compared to vehicle treated animals. These effects were not related to changes in systolic BP over the 7-day time course. This supports previous studies that have demonstrated that central administration of Ang-(1-7) induces a neuroprotective effect following ischaemic stroke, however, it is the first to investigate the longitudinal effects on lesion evolution^15,17,19,20^.

Neurological score at day 7 was not significantly different between groups an effect not surprising given the lack of difference in final infarct volume at day 7. Ang-(1-7) did not lead to any significant improvement in neurological score as assessed by the Garcia 18-point score. This test focuses on symmetry of movement, spontaneous activity and response to touch rather than provide, on its own, a comprehensive assessment of neurological deficit and behavioural outcome. In future studies, behavioural tests such as adhesive tape removal or rotarod test may provide a more robust evaluation. Regarding infarct volume, previous studies in the ET-1 induced MCAO model showed that Ang-(1-7) administered ICV as a pre and post stroke onset therapy led to a 50% reduction in infarct volume however these studies measured infarct at a single time point after treatment^15,17^. In the present study, we have demonstrated that Ang-(1-7) treatment resulted in a moderate but significant increase in tissue salvage, an effect that would have been missed without longitudinal imaging. This highlights the importance of early DWI MRI before treatment when conducting neuroprotective studies in experimental stroke studies in order to avoid type I and type II errors^29^.

At day 7 post MCAO AT_1_R mRNA expression was unchanged however, AT_2_R mRNA was upregulated compared to sham while MasR levels were significantly downregulated following MCAO. Importantly, central infusion of Ang-(1-7) following MCAO had no effect on RAS component mRNA expression when compared to vehicle treated animals. There is growing evidence that suggests activation of the AT_2_R following ischaemic stroke may have a neuroprotective role and that Ang-(1-7) may be able to act at the AT_2_R in addition to MasR^17,30^. In this study, Ang-(1-7) did not alter AT_2_R mRNA levels compared to vehicle or sham animals; however, given previously published reports, it is possible that Ang-(1-7) may have exerted some of its effects through the AT_2_R at day 7 post MCAO. In future, the use of specific antagonist studies and/or MasR/AT_2_R KO models would allow elucidate the receptor level at which Ang-(1-7) may act at different stages of injury post MCAO.

Recent findings have implicated the ‘classical RAS axis’ in mediating BBB breakdown following MCAO^31,32^. It has been suggested that central administration of Ang-(1-7) can act to preserve the integrity of the BBB at 24 hr MCAO by decreasing MMP9 and increasing TIMP1 mRNA and protein expression^23^. In contrast, we demonstrated that Ang-(1-7) ICV treatment does not influence BBB breakdown at 24 hr post MCAO nor did it alter MMP9 or TIMP1 gene expression. Furthermore, hemispheric swelling, infarct volume and neurological score were comparable between MCAO groups, indicating that at 24 hr post MCAO, Ang-(1-7) does not exert a therapeutic effect. It is important to note that in the study by Wu and colleagues, Ang-(1-7) prevented BBB disruption at lower doses used in the present study (0.5 pmol/hr and 5 pmol/hr ICV doses vs 1.1 nmol/hr) (Wu *et al*., 2015). Thus, one could postulate that a dose dependent effect may exist perhaps with a U-shaped dose response curve. Still, we demonstrated that RAS components (i.e receptor and enzyme mRNA expression) were unaltered at 24 hr compared to sham operated rats in contrast to day 7 post MCAO. This demonstrates a biphasic expression of RAS components following MCAO and may suggest that this system may not be highly involved in cerebral injury at 24 hr reperfusion.

An interesting feature observed was that the majority of rats display low levels of Gd-DTPA uptake. The BBB dynamics are under debate with reports suggesting that BBB breakdown is a continuous and long-lasting mechanism^33–35^ whilst others indicating that the BBB follows a biphasic pattern following MCAO^36–38^. The present study implies that the BBB may follow a biphasic pattern and therefore, the time point selected may not have been optimal to examine the treatment effect of Ang-(1-7) on maximal BBB breakdown. Longitudinal experiments investigating the impact of Ang-(1-7) on BBB disruption should be performed to identify the appropriate time at which maximal barrier disruption occurs and whether this is influenced by treatment.

Currently, the cellular locus at which Ang-(1-7) acts is unknown with some studies suggesting a direct anti-inflammatory effect by acting on microglia^15,17,18^. In contrast, immunohistochemical assessments performed here suggest that Ang-(1-7) did not alter IBA1^+^ microglia activation or number within the contralateral and peri-infarct regions at both 24 hr and 7 days post MCAO. Furthermore gene expression for pro-inflammatory M1 markers iNOS, IL-1β, IL-6, CD11b, NF-κsB and anti-inflammatory M2 markers CCL22, Arg1, CD163, TGF-β1 were not altered by Ang-(1-7) at the time points assessed. Although it cannot be excluded that Ang-(1-7) may exert an anti-inflammatory effect at the microglia level at 72 hr as previously hypothesised^17^, we propose that Ang-(1-7) may exert a neuroprotective effect at subacute stages of injury through Nox1 and/or CBF modulation.

At day 7, Nox1 mRNA levels were decreased in control rats compared to sham; however, this decrease was significantly attenuated in Ang-(1-7) treated rats. Previous reports have demonstrated that Nox1 KO mice have significantly increased infarct volume following MCAO when compared to wild type (WT) mice^39^. The role of Nox1 in mediating neuroprotection following stroke is not well understood. Consequently, further studies should be conducted to address its impact following MCAO and a potential Ang-(1-7) effect. A caveat in the present study is the lack of protein level confirmation. To test the neurogenesis hypothesis, immunohistochemical co-localisation analysis of Nox1 and proliferating cell nuclear antigen antibodies in the peri-infarct region would have to be performed in future. Additionally, Nox1/2 activation assays would provide a more accurate method to study the effects of Ang-(1-7) on these enzymes.

Apart from its expression in microglia and neurons, the MasR is also present in brain endothelial cells^15,40,41^. The effect of Ang-(1-7) on the cerebrovasculature has been studied with reports suggesting a vasodilatory effect in isolated rat MCA’s^42^ and vascular coronary vessels^43^. Concomitantly, in *in vivo* experiments, Ang-(1-7) ICV therapy following transient MCAO was shown to increase the potent vasodilators NO and bradykinin in the ischaemic cortex^44^ whereas in permanent MCAO models, Ang-(1-7) enhanced angiogenesis and CBF within the ischaemic penumbra^20^. In the present study, the use of LSCI allowed us to study cortical cerebral perfusion dynamics during MCAO and following reperfusion with a high temporal and spatial resolution^45,46^. Ang-(1-7) was administered immediately following reperfusion as an IV infusion at a dose set 5 times higher than the one used in ICV studies to allow cortical perfusion assessment and maximise endothelial cell MasR interaction. During reperfusion, it was observed that perfusion within the contralateral hemisphere increased over time in vehicle treated animals while Ang-(1-7) infusion significantly attenuated this increase in perfusion. In the clinic, it is reported that a shift in blood flow to non-ischaemic areas (‘steal phenomena’) is associated with worsened neurological outcome in stroke patients^47^. In addition, the degree of perfusion enhancement in the contralateral hemisphere correlates with the grade of cerebral vessel stenosis^48^. Ang-(1-7) could be acting by reversing this ‘steal phenomena’ and thereby maintaining perfusion in the ischaemic hemisphere.

Ang-(1-7) did not significantly increase perfusion within the ischaemic core or ischaemic penumbra ROIs in the present study however there was a trend towards increased perfusion within the ischaemic core territory. In permanent models, Ang-(1-7) ICV pre-treatment for 4 weeks increased CBF at 1 hour and 24 hr permanent MCAO^20^. Consequently, Ang-(1-7) may act in a cumulative manner and longer, direct treatment schedules might be necessary to observe a cerebrovascular effect in the injured ipsilateral hemisphere.

It is important to note that a major limitation with delivery of Ang-(1-7) is the short half-life of 10–20 sec and the fact that it must be administered centrally to exert a neuroprotective effect, which is not clinically feasible^49–51^. Furthermore, Ang-(1-7) induced a moderate neuroprotective effect, suggesting that the dose tested may not be optimal. Currently, novel cyclic Ang-(1-7) analogues are available and shown to be stable, long-lasting and ACE resistant^52^. Recent evidence indicates that alternative Ang-(1-7) formulations administered orally and following ET-1 induced MCAO have the potential to provide neuroprotection^53^. Therefore, to consider Ang-(1-7) as a potential thrombectomy adjuvant therapy following ischaemic stroke, new drug formulations must be tested through alternative delivery methods and dose efficacy determined.

In conclusion, this study demonstrates that the RAS is implicated in cerebral injury in a biphasic pattern with Ang-(1-7) as a post stroke therapy inducing a mild to moderate neuroprotective effect at 7 days reperfusion. Ang-(1-7) did not exert its effect via an anti-inflammatory mechanism nor prevent BBB breakdown at 24 hr reperfusion. Instead, it is hypothesised that Ang-(1-7) may exert its effects by enhancing Nox1 expression/or CBF modulation after stroke onset. Further studies must be performed to evaluate the therapeutic potential and mechanism of action of specific MasR agonists as an adjuvant therapy for thrombectomy procedures.

## Materials and Methods

### Animals and experimental design

All studies were carried out under a UK Home Office Project License, in accordance with the Animals (Scientific Procedures) Act 1986 and approved by the University of Glasgow Ethical Review Panel. A total of 97 Male Wistar rats (300–380 g) were obtained from Charles River Laboratories (Kent, UK), group housed until experimental day and single housed during recovery periods. Rats had *ad libitum* access to water and standard chow and were maintained in a controlled environment with 12:12 hour light/dark cycle and room temperature between 15–25 °C. All animals were randomly allocated to treatment groups through a list randomiser (www.random.org) prior to study commencement and investigators were blinded to treatment until data analyses were completed. For all *in vivo* experiments, sample size calculations were performed (Supplementary section). Study outcomes are reported according to the ARRIVE guidelines (http://www.nc3rs.org.uk/arrive).

Three distinct studies were conducted and the experimental protocols are described in detail in the supplementary section. The studies had the following aims:

- **Study 1**. To determine the effect of reperfusion with or without Ang-(1-7) on the extent of tissue salvage 7 days following MCAO.
- **Study 2**. To determine the effect of central administration of Ang-(1-7) on early BBB breakdown 24 hr post MCAO.
- **Study 3**. To determine whether systemic administration of Ang-(1-7) has any direct effects on the cerebrovasculature.

### Anaesthesia, analgesia and euthanasia

See supplementary methods.

### Middle Cerebral Artery Occlusion

Left MCAO was performed using intraluminal filament model as previously described^54,55^. Briefly, an arteriotomy was performed in the common carotid artery and a 4–0 nylon silicone coated tip monofilament (403934PK10 or 404134PK10 depending on rat weight; Doccol Corporation, MA, USA) inserted through the internal cerebral artery until it blocked the origin of the MCA. The filament was left in place for 90 min after which it was removed to induce reperfusion and animals were recovered from anaesthesia. For sham treated animals, vessel isolation was performed; however, the Doccol filament was not introduced.

### Intracerebroventricular delivery of Angiotensin-(1-7)

Vehicle (aCSF) or Ang-(1-7) (Bachem, Switzerland) was administered ICV as a continuous infusion via the implantation of an osmotic pump (Model 2001, ALZET, USA). A burr hole was drilled on the skull using a dental drill. An MRI compatible ICV cannula (PlasticsOne, Virginia, USA) attached to a brain infusion kit (ALZET) was inserted into the lateral ventricle (+1.6 mm lateral & −0.9 mm posterior to Bregma) and glued in place. The osmotic pump connected was inserted in a subcutaneous pocket situated posterior to scapulae.

### MRI scanning & Image analysis

All MRI data were acquired using a Bruker Pharmascan 7 T system (Ettlingen, Germany) equipped with a 4-channel phased array rat brain surface coil and a 72 mm birdcage resonator for brain imaging.

### Assessment of lesion volume and extent of tissue salvage

Diffusion Weighted Imaging (DWI) (TE = 22 ms, TR = 4 s, matrix = 96 × 96, field of view (FOV) = 25 × 25 mm, B values = 0 & 1,000 s/mm^2^, 8 contiguous coronal levels, 1.5 mm thickness, spatial resolution = 260 μm) was carried out at 30 & 60 min MCAO to generate quantitative apparent diffusion coefficient (ADC) maps and allow assessment of baseline lesion volume. Quantitative ADC maps (x10^−3^ mm^2^/s) were generated in Paravision 5 software and subsequently processed using Image J software. ADC maps were thresholded according to previously published thresholds (<0.58 × 10^−3^ mm^2^/s) from our group and lesion volume calculated by summing the area multiplied by slice thickness (Baskerville *et al*., 2016). No correction for oedema is necessary at the acute stage post MCAO and this was confirmed by measuring hemisphere volumes on ADC images.

RARE T_2_ weighted sequence (TE = 100 ms, TR = 6,000 ms, matrix = 256 × 256, FOV = 25 × 25 mm, 16 contiguous coronal slices, 0.75 mm slice thickness, in-plane resolution = 98 μm) was carried out for determination of infarct volume (24 hr or day 7) and to confirm ICV cannula placement. Infarct volume was calculated by manually delineating the hyperintense regions on T_2_ weighted images using Image J software. The lesions across the 16 coronal sections were summed, multipled by slice thickness and corrected for oedema (Gerriets *et al*., 2004).

For study 1, the extent of tissue salvage following reperfusion with vehicle or Ang-(1-7) treatment was calculated by expressing the % change in infarct volume at day 7 from the baseline lesion volume at 60 min post MCAO in the same rat.

### Confirmation of MCAO and reperfusion

During MCAO and at 24 hr and 7 days post-reperfusion, an magnetic resonance angiogram (MRA) sequence (TE = 3.8 ms, TR = 15 ms, matrix = 256 × 256, FOV = 4 × 4 mm, 50 contiguous coronal slices, 0.4 mm slice thickness, spatial resolution of 156 μm) was carried out to confirm MCAO and reperfusion. MRA data was visually assessed for the absence and presence of MCA patency.

### Assessment of BBB breakdown

To examine BBB breakdown, RARE T_1_ weighted imaging (TE = 13.5 ms, TR = 800 ms, matrix = 256 × 256, FOV = 30 × 30 mm, 8 coronal slices, slice thickness of 1.5 mm, in a plane resolution of 117 μM) was carried out prior to and post Gd-DTPA injection at 5, 10, 15, 20, 25 and 30 min from injection. RARE-T_1_ imaging analysis was conducted using an in-house Matlab code (MathWorks Ltd, UK). % signal change maps were generated by subtracting MRI-T_1_ images before and after Gd-DTPA injection and normalising to pre-contrast MRI-T_1_ scan. To determine Gd-DTPA uptake volume (mm^3^) within the ipsilateral hemisphere, the cerebral ventricles were excluded from analysis and a binary mask generated where values below 5 times the noise to signal ratio (25%) threshold were set as 0 and values above the threshold set as 1. The voxels defined as 1 were counted and multiplied by the voxel size to obtain the overall Gd-DTPA uptake brain volume (mm^3^) for each of the 6-time point scans. A final Gd-DTPA uptake volume was obtained by averaging the volumes determined in each 6-time point % signal change map.

### Laser Speckle Contrast Imaging

LSCI was carried out using a PeriCam PSI System (Perimed, Sweden) according to manufacturer’s instructions. Speckle patterns were generated and recorded at a frame rate of 10 images per sec and averaged to produce an effective frame rate of 1 image every 5 sec. LSCI baseline images were obtained for a period of 15 min during MCAO (prior to reperfusion) and continued for 90 min following reperfusion. For data analysis, perfusion images during MCAO were thresholded according to the mean perfusion in the contralateral hemisphere in order to determine tissue compartments based on perfusion thresholds (Supplementary Fig. 1). Perfusion thresholds were set to define ROIs for: 1. ischaemic core (<43% of contralateral baseline perfusion), 2. ischaemic penumbra (43–75% of contralateral baseline perfusion) during MCAO & 3. contralateral hemisphere^56,57^. For each individual ROI the baseline perfusion was calculated over 15 min during MCAO and subsequent values were then normalised to the mean signal during baseline. This allowed us to calculate the % change in cerebral perfusion following reperfusion with or without treatment. The occurrence of peri-infarct depolarisations (PIDs), which resulted in waves of hyperaemia propagating across the cortical surface, were observed and counted for each animal.

### Statistical analyses

Ischaemic lesion, percentage (%) ischaemic lesion change from MCAO, Gd-DTPA enhancement volume, % hemispheric swelling, IBA1^+^ microglia number/phenotype and systolic BP data were compared between treatment groups using unpaired Student’s t test. Gene expression data were compared between Sham, MCAO-Vehicle (aCSF) and MCAO-Ang-(1-7) groups using one-way ANOVA with Tukey’s post-hoc test. LSCI and MABP data were analysed using area under the curve (AUC) over the course of 90 min reperfusion and means compared between groups using unpaired Student’s t test. Neurological score data were compared using non-parametric Mann-Whitney test. Data were presented as mean ± S.D, shown as a scatterplot and P < 0.05 deemed statistically significant.

## Supplementary information

Supplementary Material

## Data Availability

The datasets generated during and/or analysed during the current study are available from the corresponding author on reasonable request.
