# Supplementary material for: Assessing the effects of Ang-(1-7) therapy following transient middle cerebral artery occlusion
Source: Sci Rep. 2019 Feb 28;9:3154. doi: 10.1038/s41598-019-39102-8 (PMC6395816; doi:10.1038/s41598-019-39102-8)
Supplement: Supplementary file 1 — Supplementary Material [file 41598_2019_39102_MOESM1_ESM.docx]

**Assessing the effects of Ang-(1-7) therapy following transient middle cerebral artery occlusion.**

Arroja, M.M.C^1^. Reid, E^1^. Roy, L.A^1^. Vallatos, A.V^1^. Holmes, W.M^1^. Nicklin, S.A^2^. Work*, L.M^2^. McCabe, C. ^1^

Affiliations: ^1^ Glasgow Experimental MRI Centre (GEMRIC), Institute of Neuroscience & Psychology, College of Medical, Veterinary & Life Sciences, University of Glasgow. ^2^ BHF Glasgow Cardiovascular Research Centre, Institute of Cardiovascular & Medical Sciences, College of Medical, Veterinary & Life Sciences, University of Glasgow.

***Corresponding Author:**

Lorraine Work

BHF Glasgow Cardiovascular Research Centre,

Institute of Cardiovascular & Medical Sciences,

College of Medical & Veterinary Life Sciences,

University of Glasgow,

126 University Place

Glasgow, G12 8TA

Correspondence to [Lorraine.Work@glasgow.ac.uk](mailto:Lorraine.Work@glasgow.ac.uk)

**Supplementary material**

**Sample size calculations**

All sample sizes were determined using power analysis programme G*Power (version 4.1, Germany). An ‘*a priori*’ power analysis was performed for a t test between two independent means.

**Study 1.** Using data from previous *in house* studies assessing final infarct volume following 90 min middle cerebral artery occlusion (MCAO) with 7 days reperfusion (mean infarct volume of 170mm^3^ and a S.D. of 67mm^3^). Ang-(1-7) administered as an intracerebroventricular (ICV) infusion was previously demonstrated to induce a 50% reduction in % compared to control^1^. Therefore in order to detect an effect size of 50% reduction in infarct volume, a minimum *n* number of 11 for each group with a type I error rate (α) of 0.05 and power of 0.80 was demonstrated to be necessary.

**Study 2.** Using data from the previous study, MCAO with reperfusion induces an infarct volume of 130.6mm^3^ with a S.D. of 50.7mm^3^. Ang-(1-7) administered as an ICV infusion was reported to induce approximately 50% reduction in infarcted tissue at 24 hr post stroke onset^2^. To detect an effect size of 50% reduction in infarct volume, a minimum *n* number of 9 for each group with a type I error rate (α) of 0.05 and power of 0.80 was obtained.

**Study 3.** Ang-(1-7) ICV infusion was shown to induce a 10% improvement in perfusion when compared to Vehicle treated animals in the peri-infarct region 1 hr after MCAO^3^. We have conducted pilot experiments using Laser Speckle Contrast Imaging (LSCI) where we have analysed the perfusion in different regions of interest (ROIs). Within the hypoperfused territory (CBF between 43-75% of normal) at 1 hr following MCAO, perfusion mean values were 68.6 and S.D. was 4 in perfusion units (PU). Using these data, a p value of 0.05 and setting power at 80%, a total sample size of 10 was determined (*n* of 5 per group) to detect changes in perfusion of at least 10% between groups.

**Anaesthesia, analgesia and euthanasia**

For surgical procedures, anaesthesia was induced with 5% isoflurane, rats were then intubated and artificially ventilated with 2-3% isoflurane in a 30:70% O_2_:NO_2_ mixture. Throughout the experimental procedures, body temperature was measured with a rectal thermometer and maintained at 37±0.5°C. Prior to surgery, 2 mg/kg ropivacaine (10 mg/mL; Naropin, GSK, UK) was administered subcutaneously (SC) in all incision sites. For recovery surgery, rats were administered 0.03 mg/kg burprenorphine hydrochloride (Vetergesic, Ceva Animal Health Ltd, UK) prior to anaesthetic recovery. At the end of the experimental protocols animals were euthanised under deep anaesthesia (5% isoflurane) and brain tissues were snap frozen for molecular analysis (studies 1&2) or perfusion fixed for histology (studies 1&2).

**Experimental protocols**

**Study 1. To determine the effect of reperfusion with or without Ang-(1-7) on the extent of tissue salvage following MCAO**

Transient MCAO (90 min) with 7 days recovery was carried out with rats randomly allocated to receive ICV infusion of either vehicle (n=23; artificial cerebrospinal fluid (aCSF); 1μL/hr) or Ang-(1–7) (n=23; 1.1 nmol; 1 μL/hr) commencing immediately following reperfusion. Conscious systolic blood pressure (BP) measured by tail cuff plethysmography and an 18 point neurological score^4^ was measured prior to MCAO and again at 7 days post-reperfusion. Immediately following MCAO, rats were placed in the MRI scanner and diffusion weighted imaging (DWI) carried out at 60 min post-MCAO in order to determine baseline lesion volumes prior to reperfusion and vehicle/Ang-(1-7) treatment. Magnetic resonance angiography (MRA) and RARE-T_2_ weighted MRI were conducted to confirm successful occlusion of the MCA and correct placement of the ICV cannula for subsequent therapy delivery. Reperfusion was induced at 90 min post MCAO by removal of the filament and connection of the osmotic pump for initiation of infusion of vehicle or Ang-(1-7) for a period of 7 days. At day 7 post-reperfusion, rats were again placed in the MRI scanner and RARE-T_2_ and MRA conducted to evaluate final infarct volume and confirm successful MCA reperfusion, respectively. Exclusion criteria: 1. Unsuccessful occlusion of the MCA at baseline (assessed by MRA) 2. No reperfusion of the MCA at day 7 (assessed by MRA) 3. ICV cannula not within the ventricle (assessed by T_2_ MRI) 4. MCA haemorrhage at baseline.

**Study 2. To determine the effect of central administration of Ang-(1-7) on early BBB breakdown**

Transient MCAO (90 min) with 24 hr recovery was carried out with rats randomly allocated to receive vehicle (n=16; (aCSF); 1 μL/hr) or Ang-(1-7) (n=17; 1.1 nmol; 1 μL/hr) immediately following reperfusion. 24 hr post MCAO, an 18 point neurological score was performed and rats anaesthesised for MRI scanning. To determine BBB breakdown, T_1_ weighted imaging was carried out pre and post intravenous administration of gadolinium-diethylenetriamine penta-acetic acid (Gd-DTPA, Magnevist, Bayer, UK). MR angiography was carried out to confirm left MCA reperfusion and RARE-T_2_ was conducted to assess infarct volume and correct placement of ICV cannula. Rats were excluded if cannula was not adequately placed or MCA not adequately reperfused.

**Study 3. To determine whether systemic administration of Ang-(1-7) has any direct effects on the cerebrovasculature**

Transient MCAO (90 min) with reperfusion for 90 min was carried out with vehicle (n=9; distilled water (dH_2_O)) or Ang-(1-7) (n=9; 5 nmol/hr) given as an IV infusion starting 10 min post-reperfusion. The left and right femoral veins and left artery were cannulated to allow drug, anaesthetic delivery and monitoring of mean arterial blood pressure and blood gases. Prior to MCAO and LSCI, a dental drill was used to uniformly thin the skull surface until the pial vessels were visualised. MCAO was induced and the animal placed in the stereotaxic frame for imaging. In order to minimise any effects of isoflurane anaesthesia on cerebrovascular autoregulation and neurovascular coupling we switched from isoflurane to α-chloralose anaesthesia once surgical procedures had been completed^5,6^. An initial IV bolus of α-chloralose (80 mg/kg) was given followed by a continuous IV infusion (40 mg/kg/hr)^7^. LSCI was started during MCAO and continued throughout reperfusion for the first 90 min. Animals were excluded if they did not have any perfusion deficit or if they failed to show reperfusion following removal of the filament.

**Neurological score**

Neurological score was performed prior and post MCAO using an 18-point neurological score as previously described^4^. The test is comprised of 6 distinct assessments examining sensory and motor impairment. Each test is given a maximum score of 3 and summed. The lowest the score, the worst is the neurological outcome.

**Tail Cuff Plethysmograhy**

Animals were acclimatised to the procedure for a period of 5 days prior to systolic BP measurement using the tail cuff method as previously described^8^. Systolic BP was measured one day prior to MCAO and 7 days post MCAO. For each measurement, 10 BP readings were obtained for each animal and used to calculate the mean systolic BP.

**Quantitative Real-Time PCR**

Total RNA was extracted from 50 mg of peri-infarct brain tissue from sham, vehicle and Ang-(1-7) treated animals at 24 hr (n=6-7 per group) or 7 days (n=7-9 per group) post MCAO using the Qiagen miRNeasy kit (Qiagen, UK), according to manufacturer’s instructions. Complementary DNA (cDNA) was generated from 1μg of total extracted RNA using the TaqMan Reverse Transcription Kit (ThermoFisher, UK). Samples were incubated at 25ºC (10 min), 48ºC (30 min), 95ºC (5 min), held at 12ºC and then stored at -20ºC. qRT-PCR reactions were performed using FAM-labelled probes (**Supplementary Table 1**; ThermoFisher, UK). Duplicates of each sample were incubated at 95ºC (15 min) followed by 40 cycles of 95ºC (15 sec) and 60ºC (1 min) using an ABI 7900 qRT-PCR machine (Applied Biosystems, UK). Results were analysed by taking the means of technical duplicates and normalised by comparison to the housekeeper gene selected. Data were presented as -ΔCt (cycle threshold) and normalized to housekeeping gene, ubiquitin C (Ubc) or hypoxanthine-guanine phosphoribosyltransferase 1 (Hrpt1) (study 1 and 2, respectively).

**Immunohistochemistry to detect IBA1^+^ microglia**

Brains collected at 24 hr (n=5-6 per group) or 7 days (n=2 per group) post reperfusion were perfusion fixed in 4% paraformaldehyde and paraffin embedded tissue cut at 5 μm slices over coronal levels 3, 4 and 5 covering the MCA territory. Briefly, sections were dewaxed, rehydrated and subjected to antigen retrieval. A blocking solution, tris-buffered saline (TBS) containing 10% normal goat serum (Vector Laboratories Ltd, UK) and 1% bovine serum albumin (BSA) (Vector Laboratories Ltd, UK) were applied for 2 hr at room temperature. Blocking buffer was then removed and IBA1 primary antibody (# ab139590, Abcam, UK) diluted in 1:250 TBS with 1% BSA was applied to the sections and incubated overnight at 4°C. After 24 hr, the secondary antibody (goat anti-chicken IgY (# A-11039; ThermoFisher, UK)) diluted in 1:500 TBS with 1% BSA was incubated for 1 hr in a dark room. Slides were rinsed in TBS and mounted using Vectashield medium containing 4’,6-diamidino-2-phenylindole (DAPI) (Vector Laboratories Ltd, UK). IBA1^+^ immunohistochemitry analysis was carried out by counting microglia within three ROIs established in each coronal level within the peri-infarct and homotopic contralateral area regions. ROIs were imaged at 40x using a light microscope (Leica Biosystems, UK) and captured using QCapture Pro 6 (QImaging, Surrey, Canada). IBA1^+^ microglia numbers were summed to provide total microglia count across each coronal level using Image J Software. Cells in the peri-infarct were classified into resting or activated through morphological assessment. Data were expressed as cell number per mm^2^ tissue across the three coronal levels and activated microglia phenotype expressed as % of total microglia number.

**
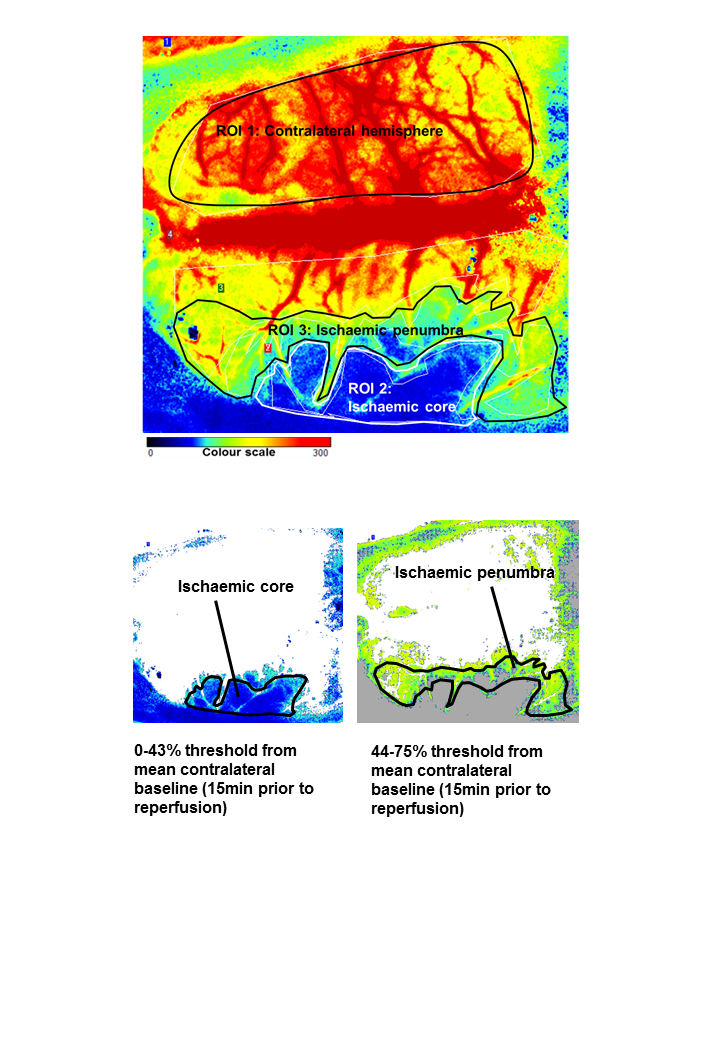
**

**Supplemental Fig 1.** Region of interest determination. A ROI was placed on the contralateral hemisphere during MCAO. From the mean PU unit obtained within the contralateral hemisphere, a threshold of 0-43% was applied to determine the ischaemic core and 44-75% threshold to establish the ischaemic penumbra.


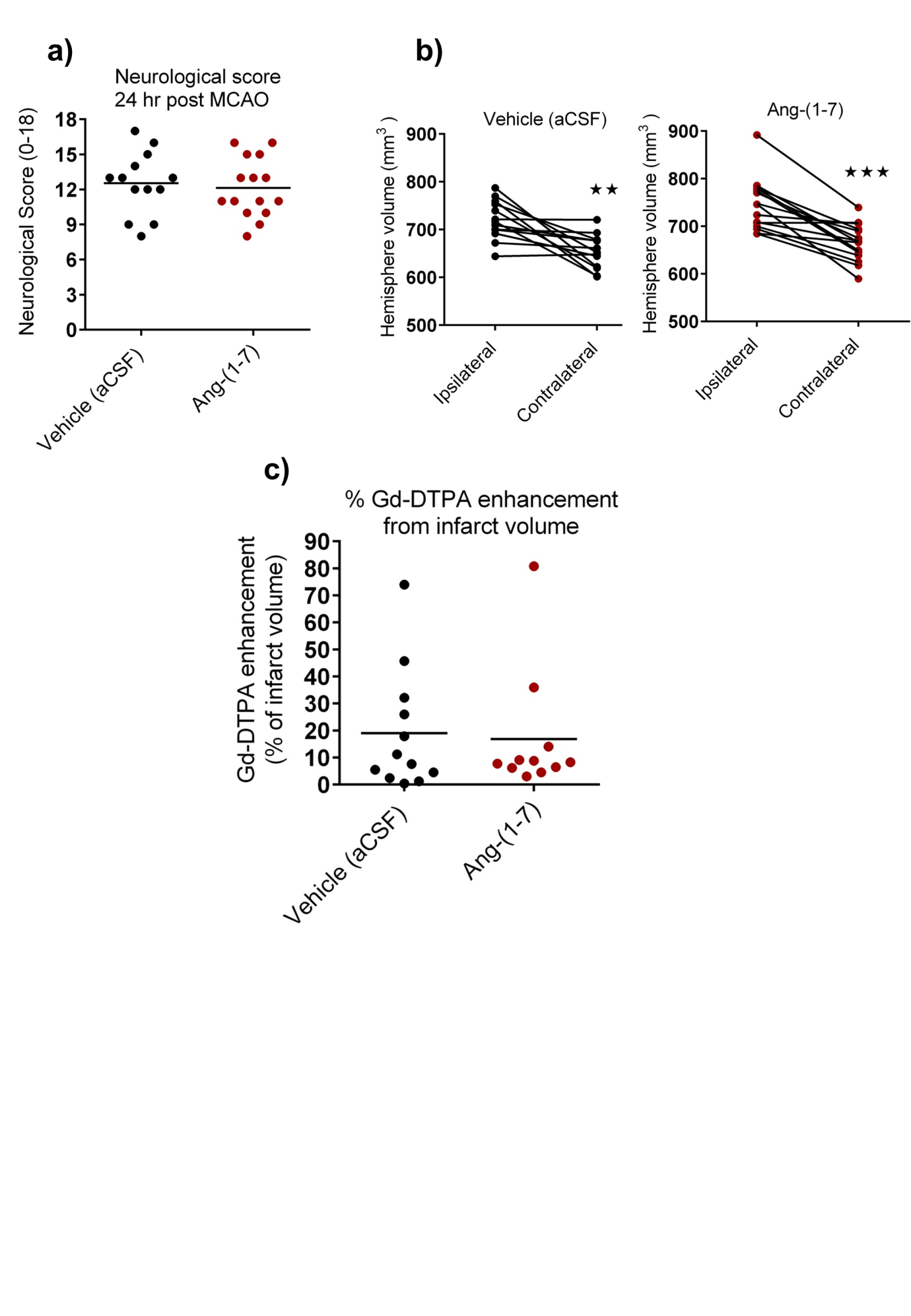


**Supplemental Fig 2. (a)** Neurological score at 24 hr post MCAO. All animals included in the study displayed signs of neurological deficit without Ang-(1-7) treated effects. **(b)** Hemispheric volume for Vehicle (aCSF) (n=13; 1 μl/hr) and Ang-(1-7) (n=15; 1.1 nmol/hr) treated rats. Ipsilateral hemisphere was significantly larger than the contralateral hemisphere for both groups, indicating BBB breakdown. **(c)** Gd-DTPA uptake expressed as percentage of infarct volume. There were no differences between treatment groups in % Gd-DTPA enhancement from infarct volume. **P<0.01; ***P<0.001; Mann-Whitney test **(a)**, paired Student’s t test **(b)** and unpaired Student’s t test **(c)**.


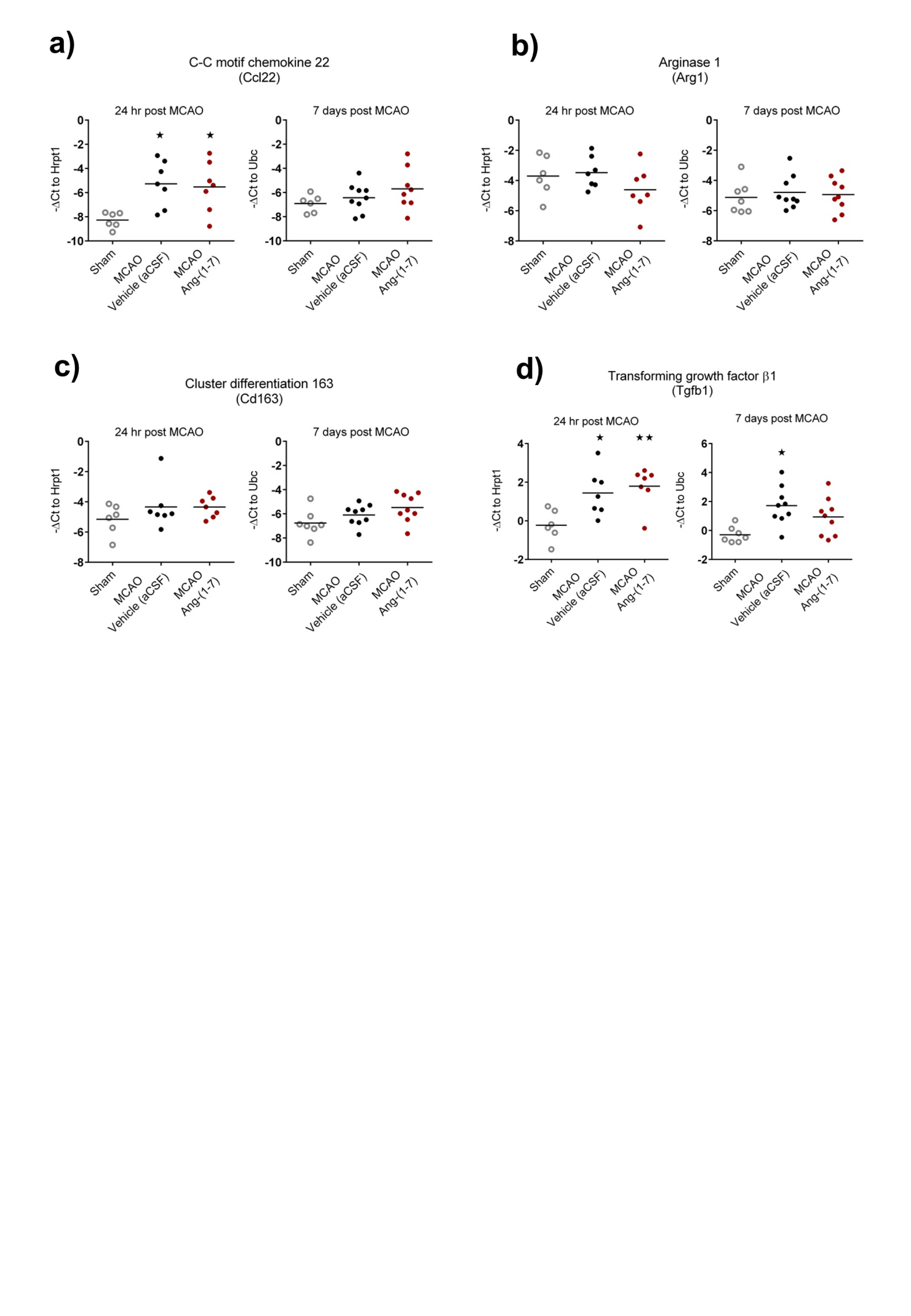


**Supplemental Fig 3.** M2 microglia/macrophage gene expression markers are not altered by Ang-(1-7) therapy following MCAO at 24 hr and 7 days reperfusion. **(a)** Ccl22 **(b)** Arg1 **(c)** Cd163 **(d)** Tgfb1. Horizontal bar represents the mean. *P<0.05; **P<0.01 compared to sham; one-way ANOVA with Tukey’s posthoc test.


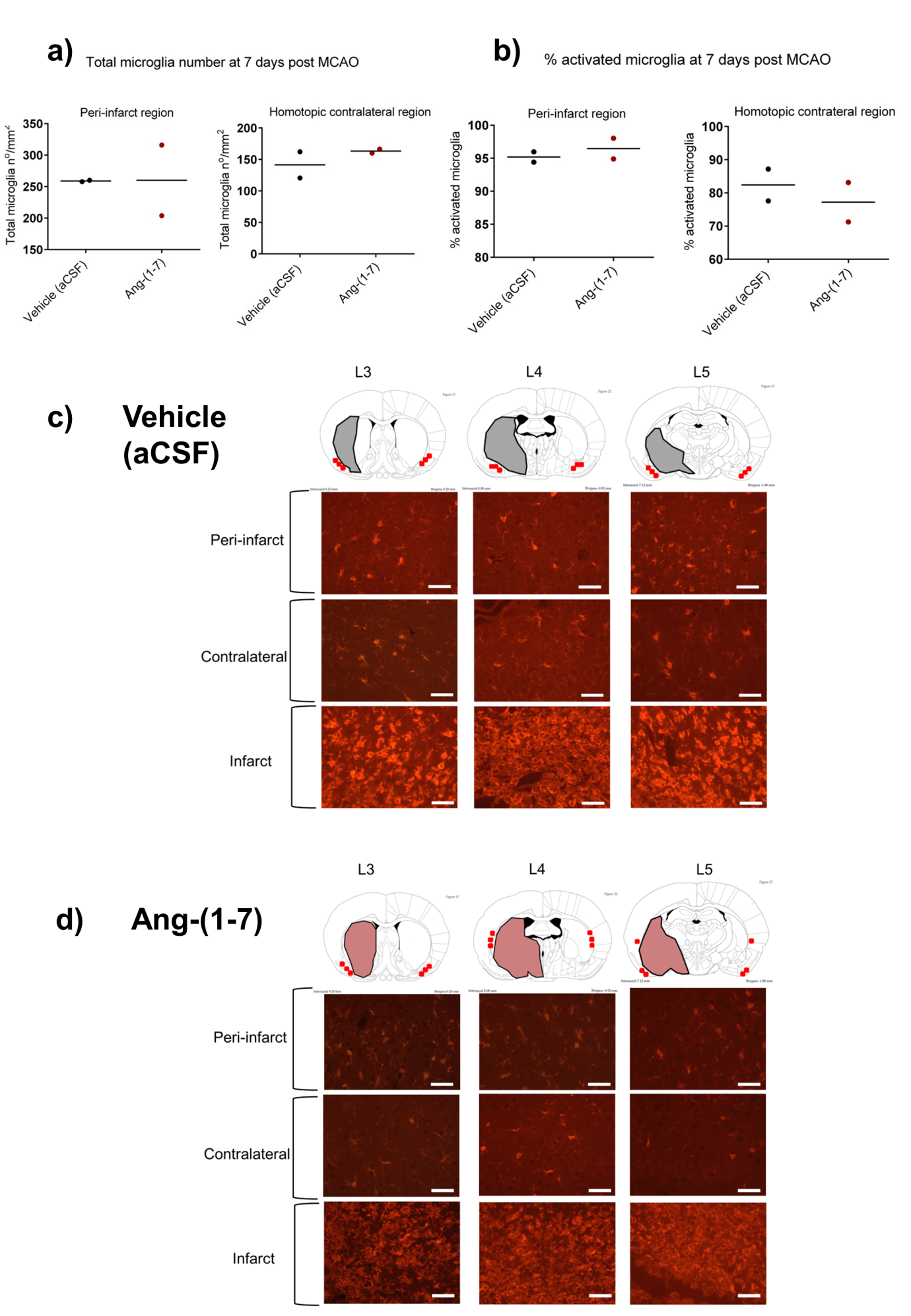


**Supplemental Fig 4.** Ang-(1-7) therapy has no effect on qualitative assessment of microglia number or phenotype 7 days post MCAO. **(a-b)** Ang-(1-7) ICV therapy (1.1 nmol/hr; n=2) did not change IBA1^+^ microglia total number or % activated cells within peri-infarct or homotopic contralateral regions compared to Vehicle (aCSF; n=2) rats. **(c-d)** Representative images of IBA1^+^ microglia staining for vehicle and Ang-(1-7) treated median animals within the peri-infarct, homotopic contralateral and infarct regions. Horizontal bar represents the mean. Line diagrams adapted from The Rat Brain in Stereotaxic Coordinates by G. Paxinos and C. Watson, 1998.


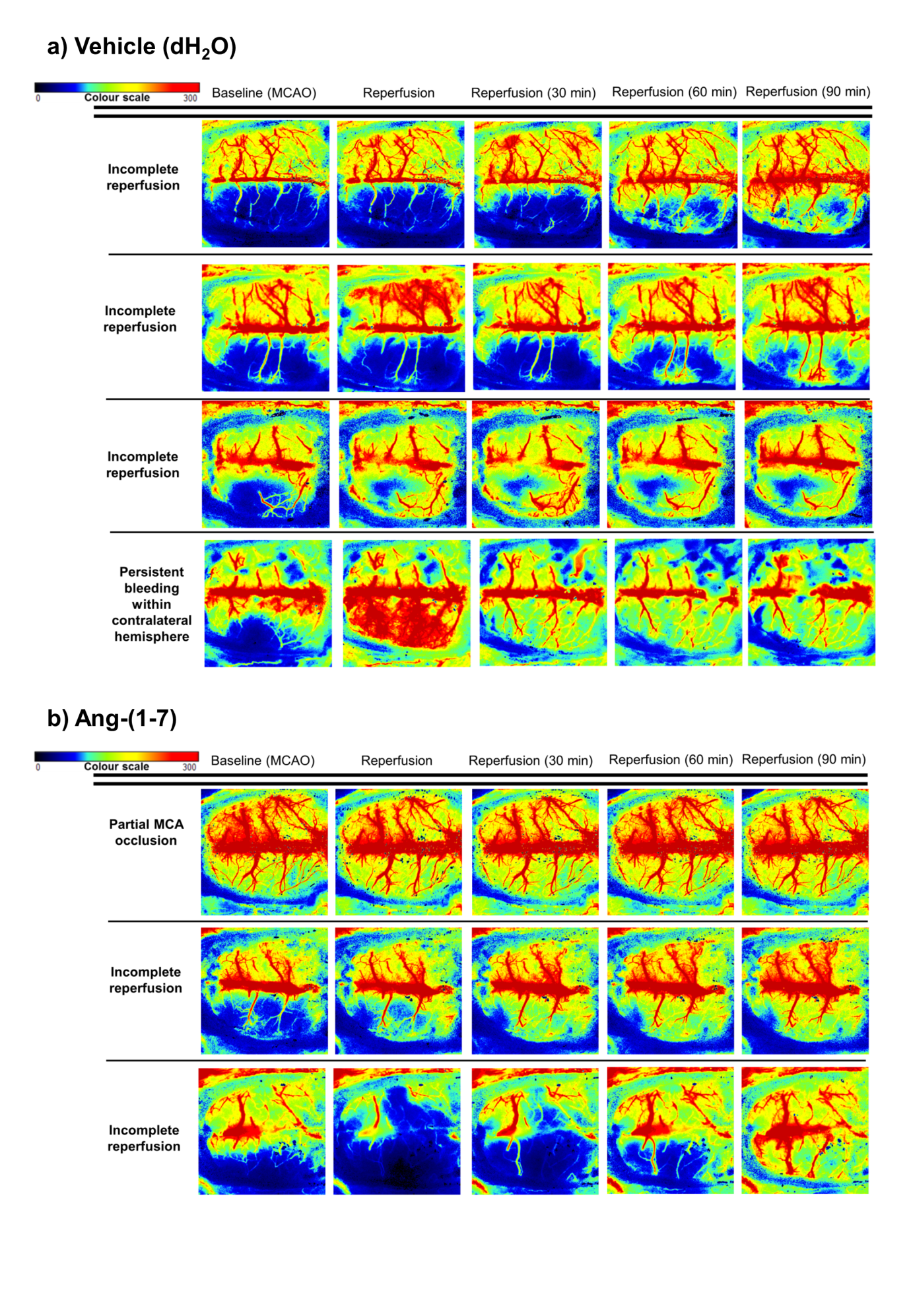


Supplemental Fig 5. LSCI study excluded animals. (a) Vehicle (dH_2_O) group (b) Ang-(1-7) group. A total of five animals were excluded due to incomplete reperfusion upon filament removal. One animal displayed signs of partial MCAO and one animal had to be excluded due to persistrent bleeding obstructing LSCI. Colour scale is set from 0 to 300 PU. A representative image of cerebral perfusion dynamics at baseline, reperfusion and every 30 min post reperfusion is shown for each animal.


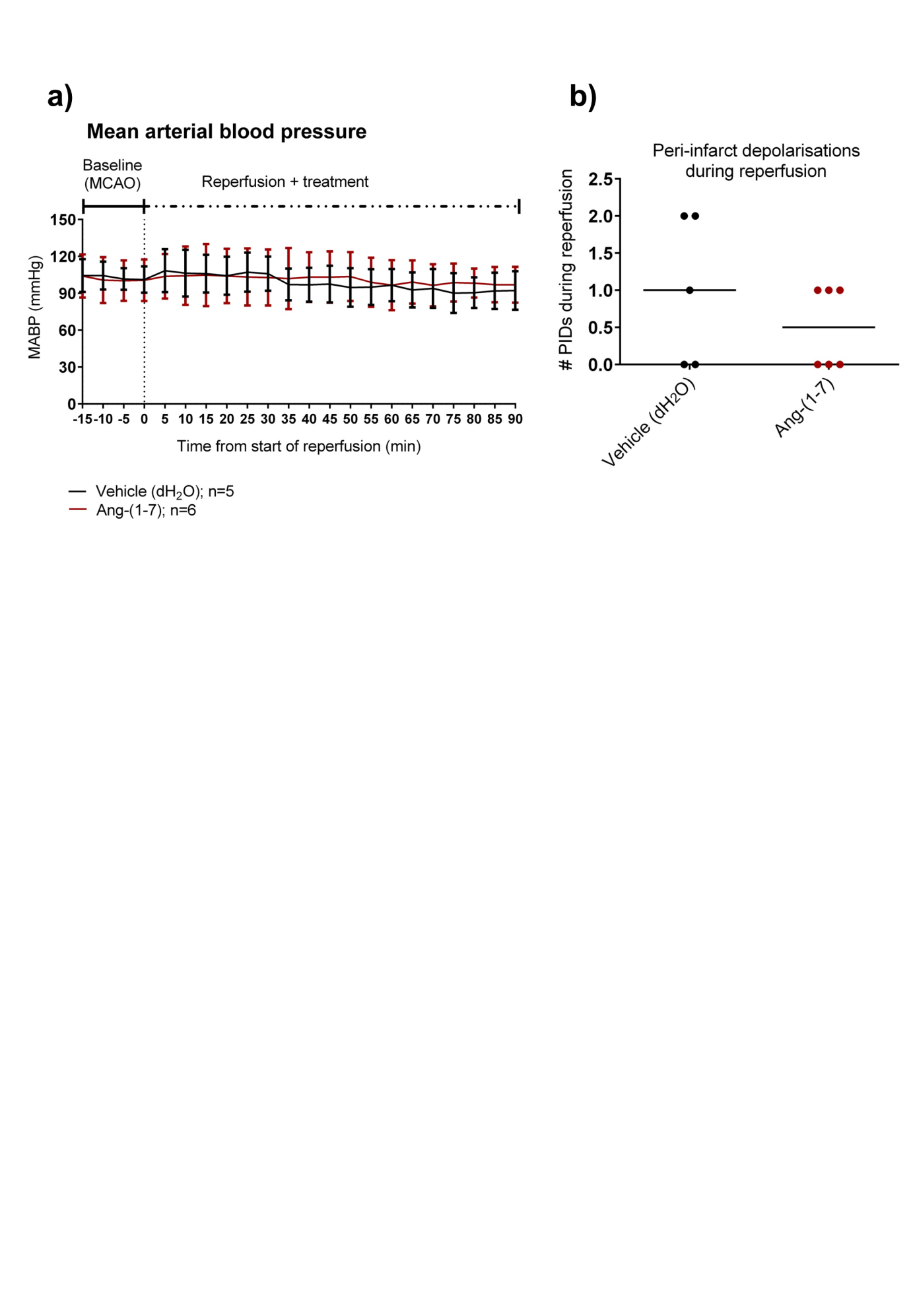


Supplemental Fig 6. (a) MABP during LSCI. Prior to therapy and during reperfusion with therapy, MABP was maintained stable between treatment groups without Ang-(1-7) induced effects. Data are expressed as MABP (mmHg) during baseline and 90 min reperfusion for vehicle (n=5; dH_~~2~~_O) and Ang-(1-7) (n=6; 5 nmol/hr) IV infusion therapy. (b) Peri-infarct depolarisation (PID) number during reperfusion. There were no differences between treatment groups in the frequency of PIDs. Data are presented as mean ± S.D for Supplementary Fig. 6a and horizontal bar represents the mean for Supplementary Fig. 6b.

|  | | **Vehicle (dH_2_O)** | **Ang-(1-7)** |
| --- | --- | --- | --- |
| **Prior to MCAO surgery** | Blood pH | 7.4±0.1 | 7.4±0.03 |
|  | Arterial PaO_2_ (mmHg) | 136.8±36.5 | 120.0±19.0 |
|  | Arterial PaCO_2_ (mmHg) | 43.5±7.2 | 41.8±2.9 |
|  | Temperature (°C) | 36.6±0.6 | 36.7±0.4 |
| **MCAO  (Baseline prior to therapy)** | Blood pH | 7.4±0.1 | 7.4±0.1 |
|  | Arterial PaO_2_ (mmHg) | 147.1±17.6 | 169.3±36.2 |
|  | Arterial PaCO_2_ (mmHg) | 36.6±6.0 | 42.3±5.5 |
|  | Temperature (°C) | 36.2±0.6 | 36.3±1.0 |
| **Reperfusion**  **(1 hr)** | Blood pH | 7.4±0.01 | 7.4±0.1 |
|  | Arterial PaO_2_ (mmHg) | 135.8±29.6 | 151.4±57.8 |
|  | Arterial PaCO_2_ (mmHg) | 42.2±3.9 | 44.4±5.0 |
|  | Temperature (°C) | 36.8±0.2 | 37.1±0.7 |
|  |  | | |

**Supplemental Table 1**. Physiological parameteres: pH, PaO_2_, PaCO_2_ and temperature prior to MCAO, at baseline and 1 hr reperfusion. Blood pH and PaCO_2_ were maintained within the normal physiological range for Vehicle (dH_2_O) and Ang-(1-7) treated groups thoroughout the experiment. PaO_2_ was above range in all experiments due to mechanical ventilation. Temperature was slightly below range 36.5°C for both groups at baseline due to animal movement between frames. Data are expressed as mean ± S.D.

| Gene | Assay ID | Refseq Gene |
| --- | --- | --- |
| Ace | Rn00561094_m1 | NM_012544.1 |
| Ace2 | Rn01416293_m1 | NM_001012006.1 |
| Agrt1a | Rn02758772_s1 | NM_030985.4 |
| Agrt2 | Rn00560677_s1 | NM_012494.3 |
| Arg1 | Rn00691090_m1 | NM_017134.3 |
| Ccl22 | Rn01536591_m1 | NM_057203.1 |
| Cd163 | Rn01492519_m1 | NM_01107887.1 |
| Hrpt1 | Rn01527840_m1 | NM_0012583.2 |
| Il10 | Rn99999012_m1 | NM_012854.2 |
| Il1b | Rn00580432_m1 | NM_031512.2 |
| Il6 | Rn01410330_m1 | NM_012589.2 |
| Itgam | Rn00709342_m1 | NM_012711.1 |
| Mas1 | Rn00562673_s1 | NM_012757.2 |
| Mmp9 | Rn00579162_m1 | NM_031055.1 |
| Nfkb1 | Rn01399572_m1 | NM_001276711.1 |
| Nos2 | Rn00561646_m1 | NM_012611.3 |
| Nox1 | Rn00586652_m1 | NM_053683.1 |
| Nox2 | Rn00576710_m1 | NM_023965.1 |
| Ptgs2 | Rn01483828_m1 | NM_017232.3 |
| Tgf1b | Rn00572010_m1 | NM_021578.2 |
| Timp1 | Rn00587558_m1 | NM_053819.1 |
| Ubc | Rn01789812_g1 | NM_017314.1 |

Supplemental Table 2. List of FAM labelled Taqman probe assays used in experimental procedures.

**References**

1. Mecca, A. P. *et al.* Cerebroprotection by angiotensin-(1-7) in endothelin-1-induced ischaemic stroke. *Exp. Physiol.* **96,** 1084–96 (2011).

2. Regenhardt, R. W. *et al.* Anti-inflammatory effects of angiotensin-(1-7) in ischemic stroke. *Neuropharmacology* **71,** 154–163 (2013).

3. Jiang, T. *et al.* Angiotensin-(1-7) induces cerebral ischaemic tolerance by promoting brain angiogenesis in a Mas/eNOS-dependent pathway. *Br. J. Pharmacol.* **171,** 4222–4232 (2014).

4. Garcia, J. H., Wagner, S., Liu, K. F. & Hu, X. J. Neurological deficit and extent of neuronal necrosis attributable to middle cerebral artery occlusion in rats. Statistical validation. *Stroke* **26,** 627–34; discussion 635 (1995).

5. Masamoto, K. & Kanno, I. Anesthesia and the quantitative evaluation of neurovascular coupling. *J. Cereb. Blood Flow Metab.* **32,** 1233–47 (2012).

6. Ueki, M., Mies, G. & Hossmann, K. A. Effect of alpha-chloralose, halothane, pentobarbital and nitrous oxide anesthesia on metabolic coupling in somatosensory cortex of rat. *Acta Anaesthesiol. Scand.* **36,** 318–22 (1992).

7. Haensel, J. X., Spain, A. & Martin, C. A systematic review of physiological methods in rodent pharmacological MRI studies. *Psychopharmacology (Berl).* **232,** 489–99 (2015).

8. Evans, A. L., Brown, W., Kenyon, C. J., Maxted, K. J. & Smith, D. C. Improved system for measuring systolic blood pressure in the conscious rat. *Med. Biol. Eng. Comput.* **32,** 101–2 (1994).
